# The ecology of avian influenza viruses in wild dabbling ducks (*Anas* spp.) in Canada

**DOI:** 10.1371/journal.pone.0176297

**Published:** 2017-05-05

**Authors:** Zsuzsanna Papp, Robert G. Clark, E. Jane Parmley, Frederick A. Leighton, Cheryl Waldner, Catherine Soos

**Affiliations:** 1 Environment and Climate Change Canada, Science and Technology Branch, Saskatoon, Saskatchewan, Canada; 2 Canadian Wildlife Health Cooperative, University of Guelph, Guelph, Ontario, Canada; 3 Department of Veterinary Pathology, University of Saskatchewan, Saskatoon, Saskatchewan, Canada; 4 Canadian Wildlife Health Cooperative, University of Saskatchewan, Saskatoon, Saskatchewan, Canada; 5 Department of Large Animal Sciences, University of Saskatchewan, Saskatoon, Saskatchewan, Canada; SWEDEN

## Abstract

Avian influenza virus (AIV) occurrence and transmission remain important wildlife and human health issues in much of the world, including in North America. Through Canada’s Inter-Agency Wild Bird Influenza Survey, close to 20,000 apparently healthy, wild dabbling ducks (of seven species) were tested for AIV between 2005 and 2011. We used these data to identify and evaluate ecological and demographic correlates of infection with low pathogenic AIVs in wild dabbling ducks (*Anas* spp.) across Canada. Generalized linear mixed effects model analyses revealed that risk of AIV infection was higher in hatch-year birds compared to adults, and was positively associated with a high proportion of hatch-year birds in the population. Males were more likely to be infected than females in British Columbia and in Eastern Provinces of Canada, but more complex relationships among age and sex cohorts were found in the Prairie Provinces. A species effect was apparent in Eastern Canada and British Columbia, where teal (*A*. *discors* and/or *A*. *carolinensis*) were less likely to be infected than mallards (*A*. *platyrhynchos*). Risk of AIV infection increased with the density of the breeding population, in both Eastern Canada and the Prairie Provinces, and lower temperatures preceding sampling were associated with a higher probability of AIV infection in Eastern Canada. Our results provide new insights into the ecological and demographic factors associated with AIV infection in waterfowl.

## Introduction

Avian influenza virus (AIV) infections remain important health issues for domestic poultry and humans in much of the world. The recent detection and rapid spread of highly pathogenic AIV (HPAIV) in North American birds (H5N8, H5N2) [1, 2], and increasing reports of outbreaks of H5N8, H5N6, H7N9, in addition to H5N1 in multiple Eurasian countries [3,4] have renewed interest in the spatial and temporal distribution, prevalence and ecology of AIV in susceptible hosts. Although several demographic and ecological determinants of AIV infection have been suggested by previous studies, risk factors remain poorly characterized and are often contradictory. Wild birds, particularly waterfowl of the order Anseriformes, are considered the natural reservoir for most subtypes of low pathogenic avian influenza viruses (LPAIV) [5,6]. Transmission of LPAIVs generally occurs through an indirect fecal-oral route involving contaminated aquatic habitats without producing overt signs of disease or mortality [5,6,7,8,9]. However, a few studies have reported associations between AIV infection and timing of migration [10], body condition [9], and resighting rates [11]. New strains can spread rapidly among individuals, H5 and H7 subtypes of LPAIV have the potential to evolve into viruses that are highly pathogenic to domestic chickens [5,12], and some strains may cause serious illness in humans [13,14,15,16,17,18,19,20]. Canada implemented an Inter-Agency Wild Bird Influenza Survey in 2005, originally with the objective of characterizing strains of AIV in Canadian wild birds and establishing a virus archive and, in 2006, was expanded to detecting highly pathogenic strains [21]. Here, we use these surveillance data to evaluate ecological and demographic risk factors associated with LPAIV infection in dabbling ducks (*Anas* spp).

In this study, we tested hypotheses about the influence of population density and local temperatures on the probability of LPAIV infection in communities of dabbling ducks while accounting for demographic, spatial and temporal effects. We asked if LPAIV prevalence is positively related to population density consistent with the hypothesis of density-dependent disease transmission, for which there is limited support [22,23]. Given that AIVs can remain infectious in water for more than 150 days depending on water temperatures [24,25], we also tested whether cooler weather around the time of sampling was associated with a higher probability of LPAIV infection, since studies have suggested an effect of environmental temperatures on AIV prevalence in wild ducks [22,26,27].

We examined the effect of sampling year on the probability of LPAIV infection to explore annual variation in LPAIV prevalence, which has been shown by other studies to exhibit a 2–4 year cyclical pattern [28,29], resulting from variation in population-level immunity and/or emergence of new strains. We also expected seasonal variation, with a peak of LPAIV infection probability during late summer or early fall [30,31,32,33,34,35] as larger flocks of birds form prior to southward migration, thereby creating greater potential for transmission.

Since juveniles tend to have a greater infection risk than adults [23,27,29,32,33,35,36,37,38], likely due to their immunologically naïve state, age was included as a covariate in all analyses. We also expected an effect of age at the population level: a duck is more likely to be exposed to AIV in populations with a higher percentage of younger, immunologically naïve ducks [26]. Evidence that infection rates differ between sexes has been contradictory [27,29,35,36,37,38,39,40]; therefore we tested whether males were more likely to be infected than females either as young or as adult birds. Based on previous large-scale North American studies [27,36,41], we hypothesized that mallards (*A*. *platyrhynchos*) would have higher infection rates than other dabbling ducks. We carried out an analysis of species effects bearing in mind the inconsistent results of previous studies [32,37,38,39,40,42].

Ours is one of only a small number of studies to have examined large scale spatiotemporal patterns and ecological determinants of AIV infection in waterfowl [22,27,35]. Our main goal was to clarify some of the environmental and ecological factors associated with LPAIV infection in wild, migratory ducks. Such analyses are essential not only for improving our understanding of AIV ecology in wild birds at continental scales, but also for enhancing future surveillance and response efforts, potentially identifying key locations and time periods for AIV infection risk.

## Materials and methods

Datasets were provided by Canada’s Inter-agency Wild Bird Influenza Survey [36]. During 2005–2011, 19692 live dabbling ducks were captured and tested for AIV at numerous sampling locations across Canada. Birds were captured, banded and sampled under the authority of Environment and Climate Change Canada–Canadian Wildlife Service Scientific Research Permit (CWS07-005), Saskatchewan Ministry of Environment Special Permit (09FW131, renewed annually), University Committee on Animal Care and Supply (UCACS), Animal Use Protocol Number (20070039), Migratory Bird Sanctuary permit (for working at Last Mountain Lake; renewed annually), National Wildlife Area permit (for working at Last Mountain Lake; renewed annually) and Environment and Climate Change Canada Migratory Bird Banding permit (Soos, 10458R). Bird capture, handling and sampling procedures were approved by the Environment and Climate Change Canada Animal Care Committee (protocol numbers 0500JK01, 0800JK01, 0929CS01, 1029CS02, 1129CS02) and the University of Saskatchewan's Animal Research Ethics Board (protocol #20070039), and adhered to the Canadian Council on Animal Care guidelines for humane animal use.

**Fig 1:**
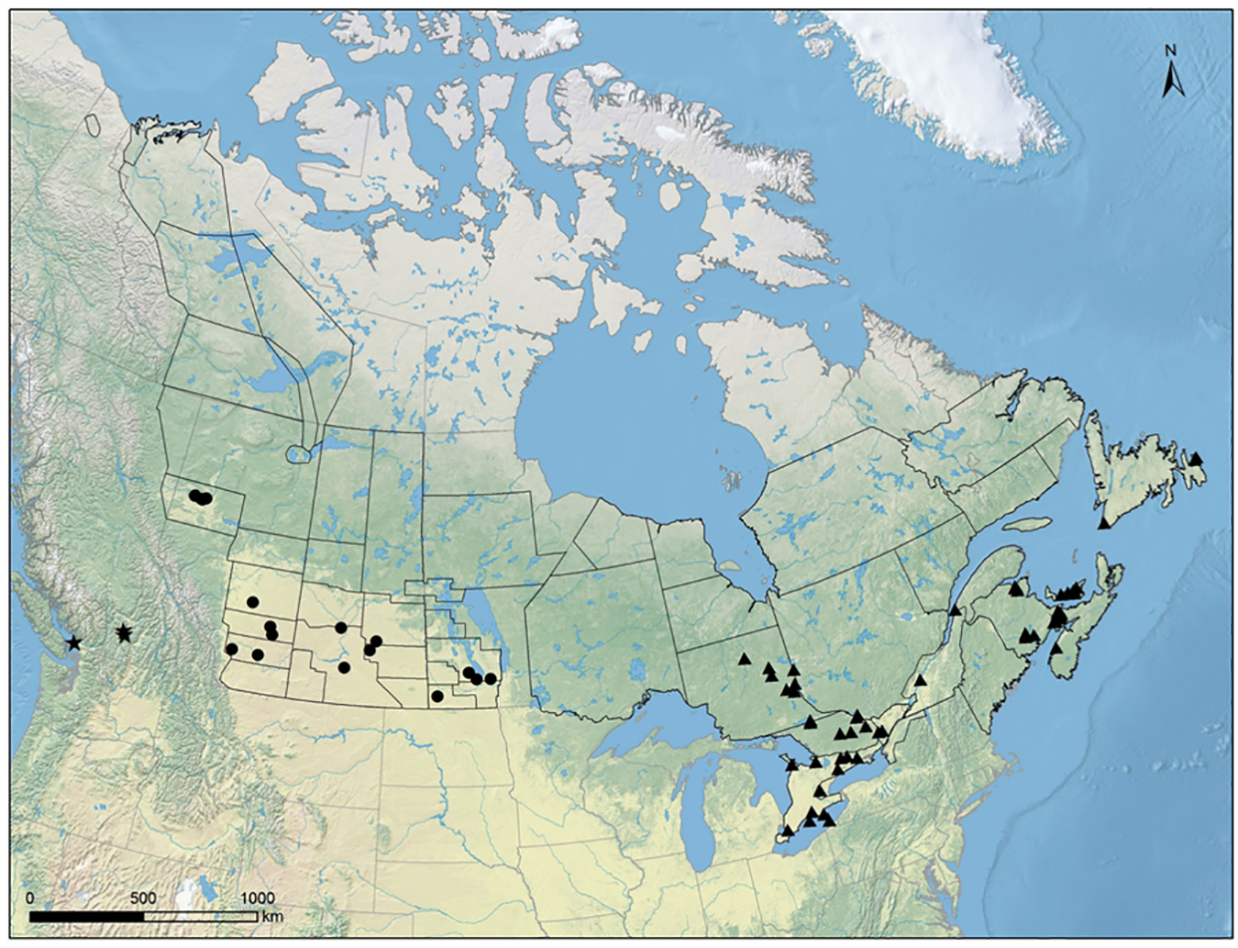
Sampling locations for *Anas* spp. for Canada’s Inter-agency Wild Bird Influenza Survey, 2005–2011. Sections indicate duck population survey strata for which population density was estimated in spring. Star: British Columbia; Round dots: Prairie Provinces; Triangles: Eastern Canada.

Samples considered for this study were obtained by capturing ducks between mid-July and late December from 95 sampling locations (Fig 1), 20 in the Prairie Provinces, 72 in Eastern Canada and three in BC. The vast majority of ducks were captured using bait traps, however netting ducks from airboats was also used as a capture method at a few sites in Eastern Canada (Soos et al, 2012). Most samples (~92%) were collected in August and September. In 2005, a single cloacal swab was obtained from each duck; thereafter, the sampling protocol involved obtaining a combined sample of oropharyngeal and cloacal swabs from each bird [21]. Samples were analyzed for the presence of the matrix protein gene segment (M1) common to all influenza A viruses using the real-time reverse transcriptase polymerase chain reaction (RRT-PCR) assay as described elsewhere [21, 36, 43]. The same method was used in all participating laboratories. A sample with a PCR threshold cycle value of 35 or lower was considered positive. Datasets included PCR results for individual birds tested, along with the full complement of field data containing all variables listed in S1 Table.

### Selection of model variables

To assess risk factors associated with AIV prevalence in dabbling ducks, data from Eastern Canada (Ontario (ON), Quebec (QC), Nova Scotia (NS), Prince Edward Island (PEI), New Brunswick (NB), Newfoundland and Labrador (NL)), the Prairie Provinces (Manitoba (MB), Saskatchewan (SK) and Alberta (AB)) and British Columbia (BC) were analysed separately because of differences in availability of explanatory variables (e.g., age, species, year, population density) or differences in field methods (e.g., ranges of sampling dates, estimation of population density). Importantly, these three breeding regions also correspond well with the main migratory Flyway affiliations of most ducks throughout their annual life-cycles, i.e., Atlantic Flyway for Eastern Canada, Mississippi and Central Flyways for Prairie Provinces and Pacific Flyway for BC [44]. The response variable was binary, representing the presence or absence of infection with any AIV (matrix positive or negative for AIV M1 gene). AIV occurrence was modelled in relation to demographic (age, sex, species), population level (population density, percent mallards, percent hatch-year (HY) birds in population), temperature and temporal (year, sampling date) factors described in S1 Table.

The overall percent HY birds was estimated for each year and hunting zone using Environment and Climate Change Canada (ECCC)'s Harvest Survey Data (http://www.ec.gc.ca/reom-mbs/enp-nhs/index.cfm?do=b&lang=e) which provides age and sex data for all migratory game bird species hunted in Canada. Information from all available dabbling duck species in each hunting zone was pooled to calculate the overall percentage of HY birds.

Since the surveillance data did not include reliable estimates of population density at the time of sampling, we obtained spring breeding population density data from the Waterfowl Breeding Population and Habitat Survey for 2005–2011 [45] for each of the regions in which ducks were sampled. Total population density was defined as the number of dabbling ducks estimated per km^2^ within each stratum surveyed during the breeding season, while pond density (an alternative correlate of population density in the Prairie Provinces dataset) was defined as the number of standing water bodies estimated per km^2^ of transects within each stratum surveyed during the breeding season [45]. We used spring breeding population density as a proxy for population density in the sampling period under the assumption that there would be a positive correlation between spring breeding density and August population density; spring population densities are positively correlated with spring pond densities [35,46] and reproductive success is generally higher in years of abundant ponds and breeding populations [47]. Population density estimates were not available for most of our samples from BC, therefore this variable was not evaluated in BC models.

Since it has been hypothesized that mallards (MALL) have a major role in the epidemiology of AIV [27,45], we calculated the percentage of mallards (% MALL) in the breeding duck population from species-specific density estimates [45] and used this as an explanatory variable for the probability of AIV infection.

Given that cooler water and air temperatures may enhance the environmental persistence of viruses, we calculated local mean daily temperature for the two week period before the sampling day (sampling day inclusive). Data were obtained from Environment and Climate Change Canada's National Climate Data and Information Archive (NCDIA) for the weather station closest to each sampling location (http://climate.weather.gc.ca/historical_data/search_historic_data_e.html), with complete or close to complete temperature information. Missing values (for up to three consecutive values) were estimated by taking the average of the values for the previous and next closest available dates. If more than three data points were missing, data from the next closest weather station were used. The mean distance between sampling locations and corresponding weather stations was 21.8 km (range of 2.7–76.8 km).

Sampling date was used to examine the variation in prevalence of AIV within year. Date of sampling was expected to have a nonlinear relationship with AIV prevalence, therefore a categorical variable “Sampling Time” was created based on two week sampling periods in the Prairie Provinces. Similar two week periods for July and August plus the months of September and October and a combined category of November-December were used with samples from Eastern Canada.

We included year as a fixed effect in all model sets to examine annual patterns of prevalence while recognizing that this may result in absorbing the annual variation caused by more biologically meaningful variables. Sampling location (i.e., lake or wetland where samples were collected) was entered as a random effect in the analysis of the prairie and Eastern Canada datasets, and as a fixed effect in the BC dataset to account for spatial clustering of data.

### Statistical modelling strategies

Individuals with missing data for sex, age, or diagnostic test results were excluded from analysis. Descriptive statistics were calculated to estimate overall apparent prevalence (proportion of infected birds).

To explain variation in AIV status, a set of models was built based on general guidelines described by Nallar et al. [23,35]. For the Prairie Provinces and Eastern Canada, associations between explanatory variables (S1 Table) and AIV infection were evaluated with generalized linear mixed effects models with binomial distribution, using sampling location as a random intercept. Models were built with the “lmer” function (“lme4” library) in R [48] using a logit link function and Laplace approximation of the maximum-likelihood. To analyse BC data, general linear models with binomial distribution were run using the “glm” routine with a logit link function, with sampling location as fixed effect.

First we explored unconditional models that included only one fixed effect predictor. Variables that improved the null model (lowered the Akaike Information Criterion, AIC) were combined in biologically meaningful ways in more complex models, while minimizing the number of models explored. Continuous variables were assessed for linearity with the outcome. We included age, sex, age-sex interaction and species in complex models based on their hypothesized importance as basic demographic explanatory variables. We then added year and sampling time followed by other predictors of interest in different combinations. Variables that were highly correlated (Pearson or phi correlation coefficient >|0.4|) were not combined in the same model [49].

Throughout the model building process we identified non-informative variables, i.e., those that did not lower the AIC_c_ (AIC, adjusted for sample size) of a model when included. We list such non-informative variables in italics in S3–S5 Tables. As the last step of the model building process, we dropped any non-informative variables from the highest ranking model, which resulted in the overall best-approximating model.

Model selection was carried out using AIC_c_ [50] to rank competing models. We considered the model with the lowest AIC_c_ to have the best support given the data.

## Results

In total, 19,157 ducks met our inclusion criteria: 8967, 7909 and 2281 for Eastern Canada, the Prairie Provinces and BC, respectively (S2 Table). Apparent prevalence of AIV infection was 25.0% overall (CI = 24.4, 25.6) with 29.4% (CI = 28.4, 30.3) in Eastern Canada, 17.6% (CI = 16.8, 18.5) in the Prairie Provinces and 33.4% (CI = 3.1, 35.4) in BC.

### Eastern Canada

**Fig 2:**
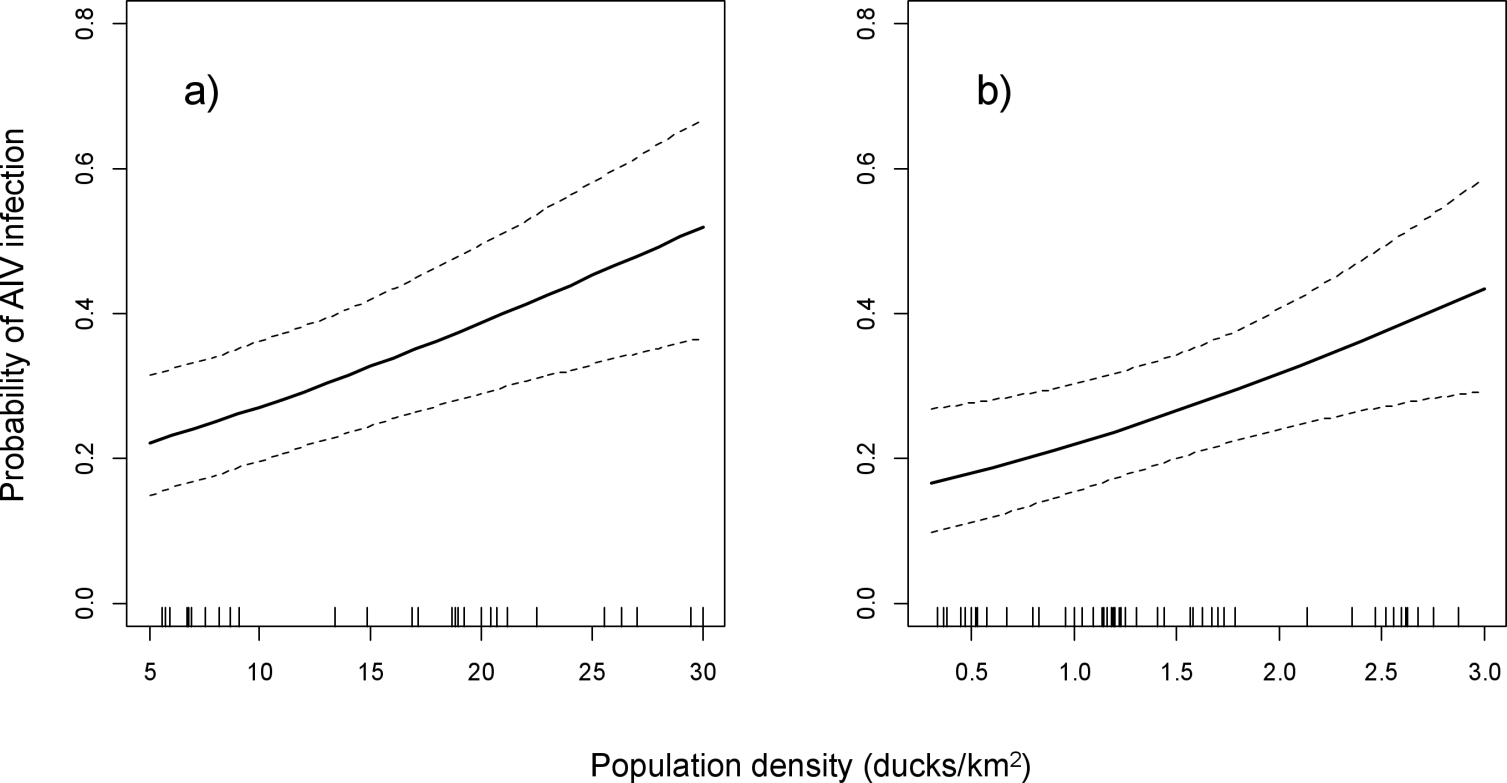
Predicted probability (solid line) and 95% CI (dashed lines) of AIV infection in dabbling ducks in the Prairie Provinces (a) and Eastern Canada (b) as a function of population density. Probabilities were estimated based on the best-approximating model (Tables 1 and 2), with other variables set to their model reference category over the dataset as shown in the reported tables (HY male ducks (Prairie) or mallards (East) in Aug 2007) and at their mean values for temperature and percent HY. The distributions of data points are presented as rug plots along x axes (i.e., a vertical bar represents each sample) with different scales because duck density is higher in the Prairie Provinces than in Eastern Canada.

Candidate models to explain variation in AIV infection status in Eastern Canada are displayed in S3 Table. Based on the best-supported model, infection status was positively associated with population density. After accounting for all other risk factors in the model, for every one duck per km^2^ increase in population density, the odds of AIV infection at the time of sampling increased 1.65 times (CI = 1.14, 2.39) (Table 1, Fig 2).

**Table 1:** Maximum likelihood estimates (with 95% CI) of covariate parameters in the best-approximating model fitted to estimate the variation in AIV infection probability in dabbling ducks (*Anas* spp.) in Eastern Canada.

Variable	Term	MLE	SE	95% CI
Intercept		-3.741	1.561	-6.800, -0.682
Age (ref = HY)	AHY	-0.785	0.103	-0.987, -0.582
Sex (ref = Female)	Male	0.124	0.054	0.017, 0.231
Species (ref = MALL)	ABDU	-0.029	0.093	-0.210, 0.153
	AGWT	-0.706	0.130	-0.962, -0.451
	AMWI	-0.399	0.173	-0.738, -0.061
	BWTE	-0.542	0.139	-0.815, -0.269
	NOPI	-0.254	0.393	-1.023, 0.516
Population density		0.503	0.188	0.134, 0.873
Temperature		-0.104	0.027	-0.157, -0.052
Percent HY		0.056	0.017	0.023, 0.090
Sampling Time (ref = July 21 –Aug 5)	Aug 6–19	0.281	0.162	-0.037, 0.599
	Aug 20–31	0.555	0.184	0.195, 0.915
	Sept 1–30	0.527	0.215	0.106, 0.947
	Oct 1–31	0.3	0.374	-0.433, 1.032
	Nov 1 –Dec 31	-1.014	0.575	-2.141, 0.112
Year (ref = 2005)	2006	-0.375	0.094	-0.560, -0.190
	2007	-1.27	0.098	-1.462, -1.078
	2008	-3.13	0.298	-3.715, -2.546
	2009	-1.909	0.168	-2.238, -1.579
	2010	-2.252	0.138	-2.523, -1.981
	2011	-1.177	0.261	-1.688, -0.666

The model was fitted as a generalized mixed effects model with sampling locations (72 sites) as a random intercept term (variance of 1.32) and other explanatory variables as fixed effects, with a sample size of 8967. All values shown are on the logit scale. Exponentiation of estimates provides the odds ratio interpretation of the effect size. MLE is the maximum likelihood estimate of the parameter, SE is the standard error of the estimate, and 2.5% and 97.5% values define the 95% confidence interval around the MLE. Population density is the total number of ducks/km^2^ at the beginning of the breeding season in the corresponding survey stratum, Temperature is the average of mean daily temperatures over the two weeks before the sampling day (sampling day inclusive) and Percent HY is the percentage of hatch year birds reported shot by hunters in the population (fall) in the corresponding hunting zone.

Abbreviations: HY: Hatch Year; AHY: After Hatch Year; MALL: *A*. *platyrhynchos*, ABDU: *A*. *rubripes*, AGWT: *A*. *carolinensis*, AMWI: *A*. *americana*, BWTE: *A*. *discors*, NOPI: *A*. *acuta*

**Table 2:** Maximum likelihood estimates of fixed effects in the best-approximating model of AIV infection in the Prairie Provinces.

Variable	Term	MLE	SE	95% CI
Intercept		-2.333	0.295	-2.910, -1.755
Age (ref = HY)	AHY	-0.878	0.145	-1.162, -0.594
Sex (ref = Female)	Male	0.113	0.076	-0.037, 0.263
Age*Sex		-0.440	0.162	-0.758, -0.123
Population density		0.053	0.015	0.023, 0.083
Sampling Time (ref = July 23 –Aug 5)	Aug 6–19	0.506	0.109	0.293, 0.719
	Aug 20 –Sept 1	-0.200	0.139	-0.473, 0.072
Year (ref = 2005)	2006	1.299	0.171	0.965, 1.634
	2007	0.188	0.145	-0.096, 0.473
	2008	-2.565	0.310	-3.173, -1.957
	2009	-0.235	0.187	-0.602, 0.132
	2010	0.947	0.169	0.616, 1.278
	2011	-0.400	0.227	-0.845, 0.044

The model was fitted as a generalized mixed effects model with sampling location (20 sites) as a random intercept term (variance of 0.665) and other explanatory variables as fixed effects, with a sample size of 7909. All values shown are on the logit scale. Exponentiation of estimates provides the odds ratio interpretation of effect size. MLE is the maximum likelihood estimate of the parameter, SE is the standard error of the estimate, and 2.5% and 97.5% values define the 95% confidence interval around the MLE. Population density is the total number of ducks/ km^2^ at the beginning of the breeding season in the corresponding survey stratum. Age*Sex is the interaction term.

Abbreviations: HY: Hatch Year; AHY: After Hatch Year

**Fig 3:**
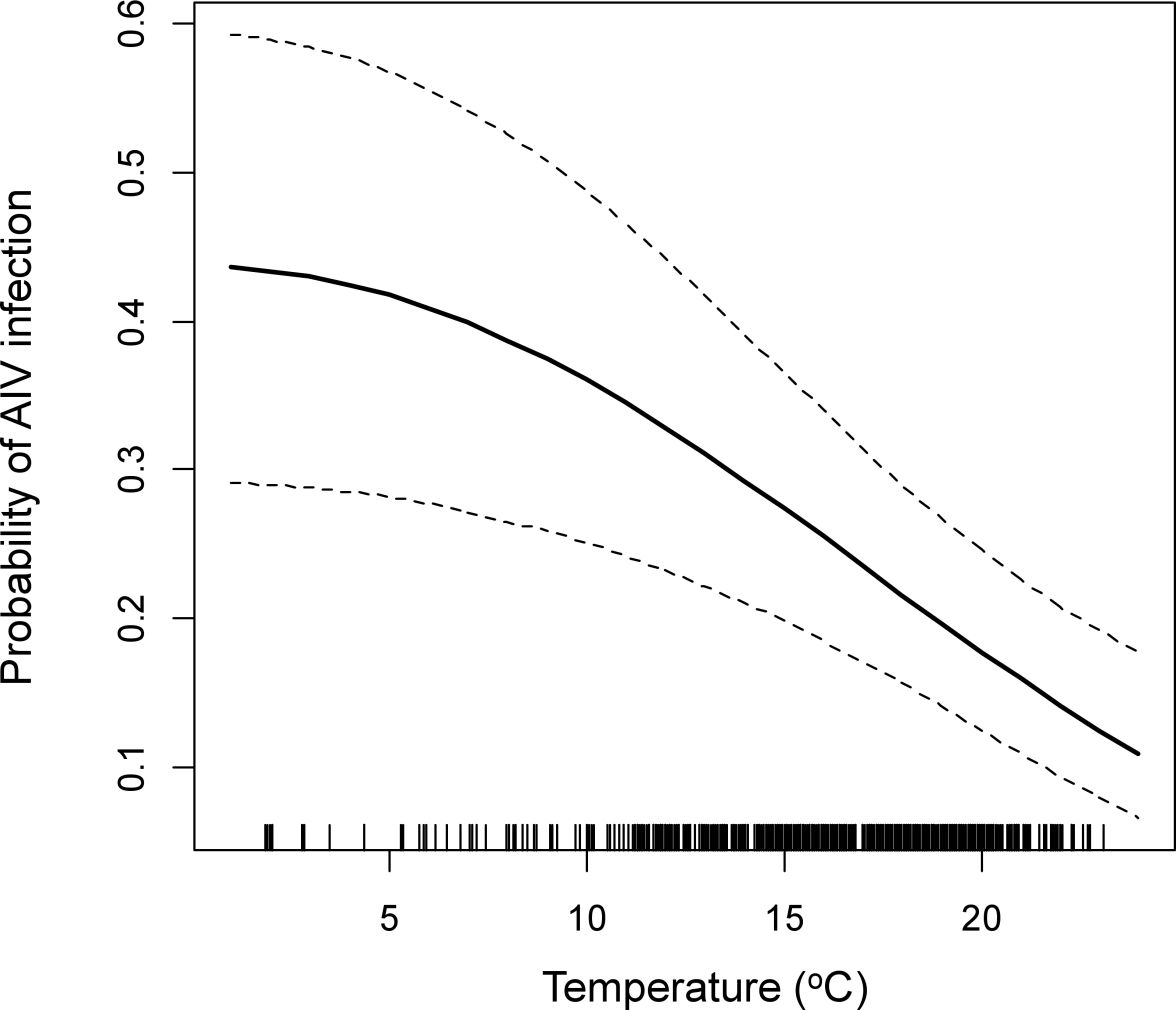
Predicted probability (solid line) and 95% CI (dashed lines) of AIV infection in dabbling ducks in Eastern Canada as a function of local mean daily temperature two weeks preceding sampling. Probabilities were estimated based on the best-supported model (Table 1), with other variables set to the reference category over the dataset (HY male mallards in Aug 2007) and at their mean values for population density and percent HY). The distribution of data points is presented as rug plots along the x axis (i.e., a vertical bar represents each sample).

**Fig 4:**
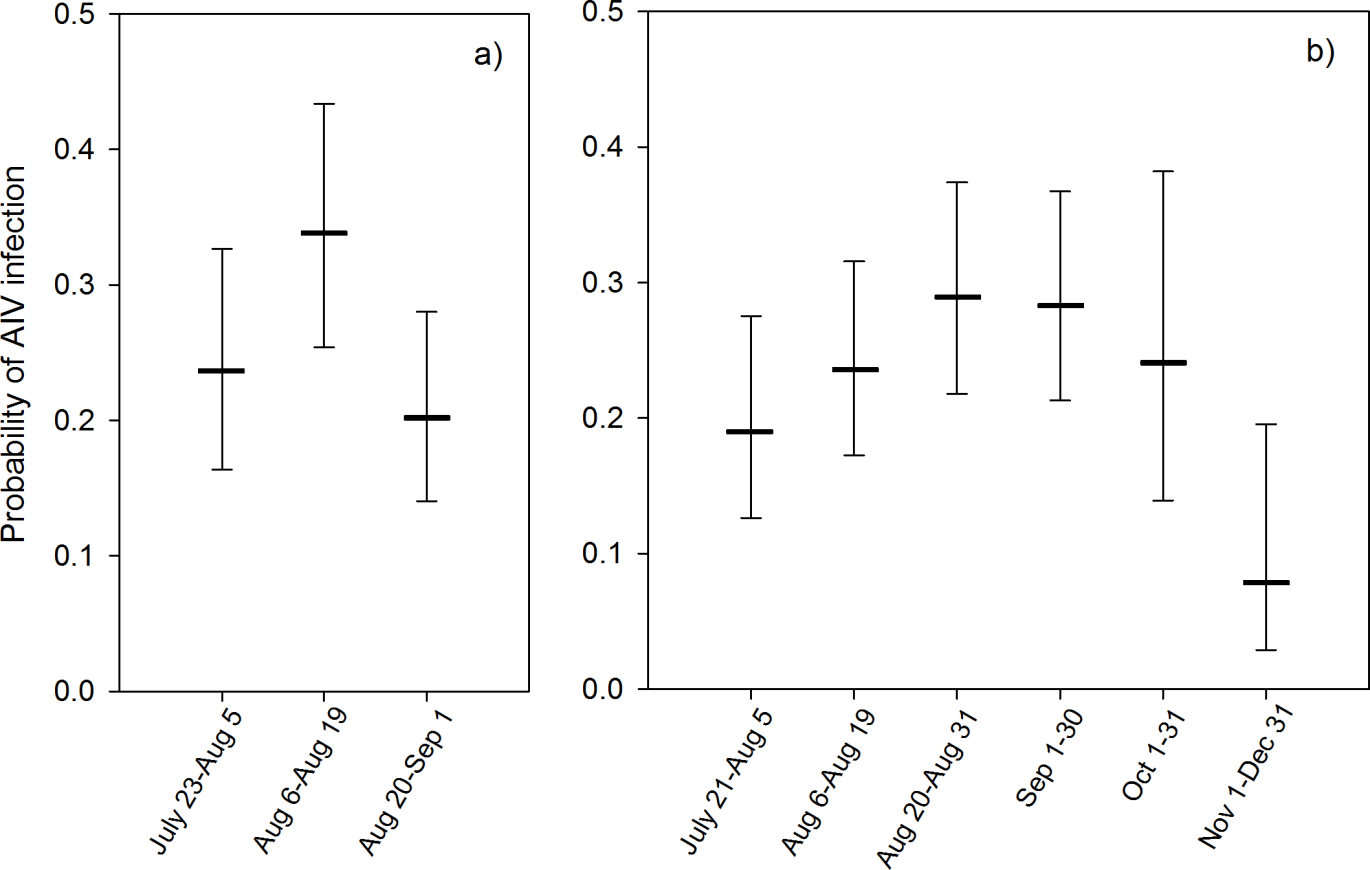
Predicted probability of AIV infection with variation in sampling time in dabbling ducks in the Prairie Provinces (a) and Eastern Canada (b). Probabilities were estimated based on the best-approximating model (Tables 1 and 2), with other variables set to the reference category over the dataset (HY male ducks (Prairie) or mallards (East) in 2007) and at their mean values for population density and percent HY. Sampling date categories represent approximately 2-week periods in the Prairie Provinces (July 23-Aug 5, Aug 6–19, Aug 20-Sept 1), and closely matching periods followed by monthly categories in Eastern Canada (July 21-Aug 5, Aug 6–19, Aug 20–31, Sept 1–30, Oct 1–31, Nov 1-Dec 31). Sample sizes for each region are shown in S2 Table.

**Fig 5:**
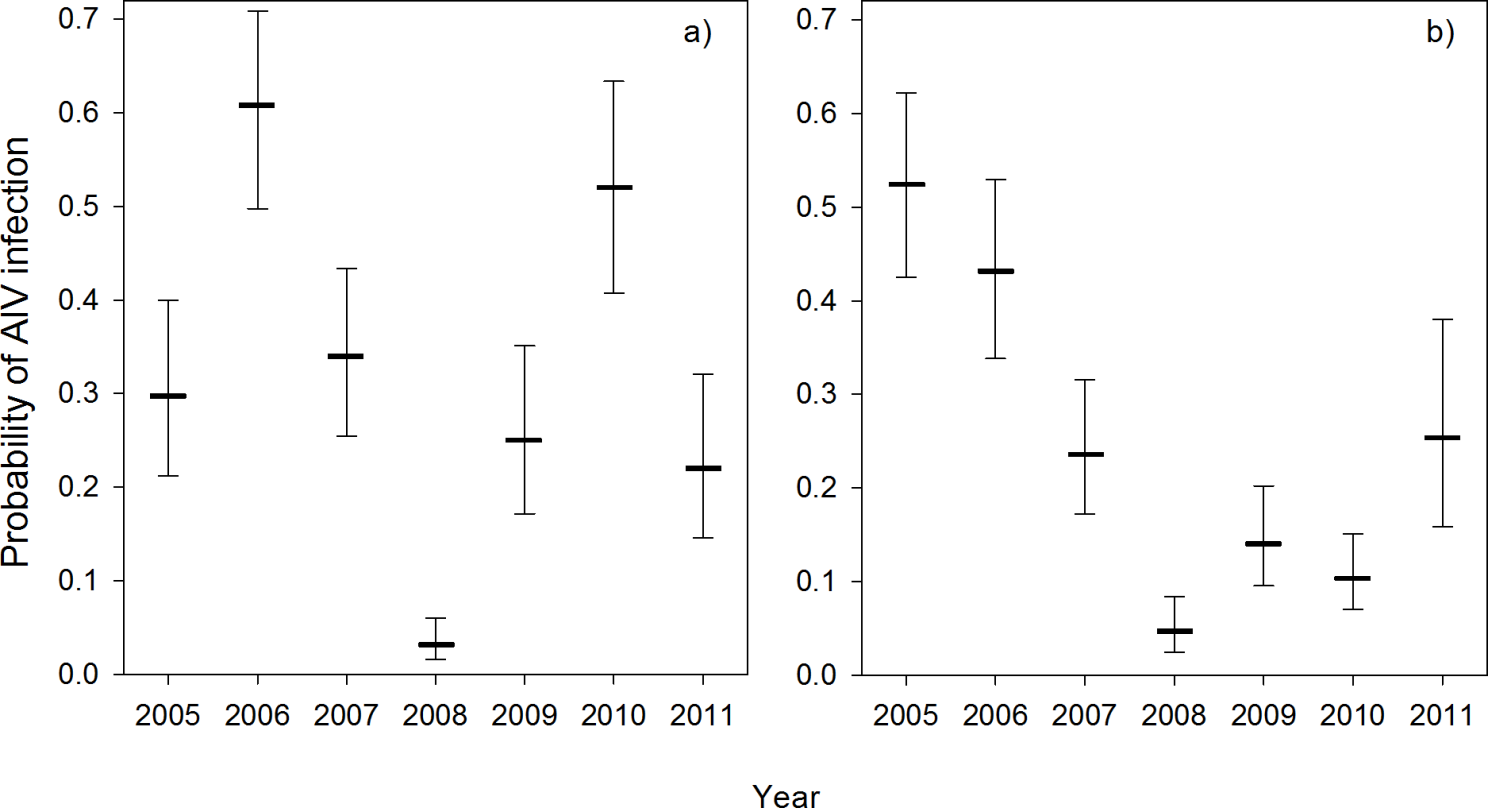
Annual variation in predicted probability of AIV infection in dabbling ducks in the Prairie Provinces (left) and Eastern Canada (right). Vertical lines represent 95% CI. Probabilities were estimated based on the best-approximating model (Tables 1 and 2), with other variables set to the reference category over the dataset (HY male ducks (Prairie) and mallards (East) in August and at their mean values for mean temperature, population density and percent HY, where applicable). Sample sizes for each region and year are shown in S2 Table.

Mean average daily temperature values during the two weeks preceding the day of sampling was negatively associated with AIV infection risk (Table 1, Fig 3). For every one °C decrease in temperature, the odds that a duck would test positive increased by 10% (odds ratio, OR = 0.9, CI = 0.85, 0.95). The probability of a duck being infected with AIV was highest in late August-September (Table 1, Fig 4). Highest and lowest estimated AIV prevalences occurred in 2005 and 2008, respectively (Fig 5). Infection status was positively associated with the percentage of HY ducks in the sampling region (Table 1). For every 10% increase in the proportion of HY ducks in the sampling region, the odds that a duck would test positive for AIV increased 1.75 times (CI = 1.26, 2.46). Young birds were more likely to be infected than adults (OR = 2.19, 95% CI = 1.79, 2.68), while males were more likely to be infected than females (OR = 1.13, CI = 1.02, 1.26) (Table 1). Blue-winged teal, green-winged teal and American wigeon were less likely to be infected than were mallards (Table 1).

### Prairie Provinces

Candidate models to explain variation in AIV infection status in the Prairie Provinces are displayed in S4 Table. Based on the best-supported model, infection status was positively associated with population density: for every 10 ducks per km^2^ increase in the population density, the odds of AIV infection at the time of sampling increased 1.7 times (CI = 1.26, 2.29) (Table 2, Fig 2). The probability that a duck was infected with AIV was highest in mid-August (Table 2, Fig 4). The probability of being infected was lowest in 2008 (Fig 5). There was an interaction between age and sex effects: among after hatch year (AHY) birds, females were 1.38 times more likely to be infected than males (CI = 1.04, 1.82) (Table 2), while there was no difference between females and males in HY birds (OR = 1.12, CI = 0.96, 1.30). HY birds were 2.41 and 3.71 times more likely to be infected than AHY females and males, respectively (CI = 1.81, 3.20 and 3.05, 4.51, respectively). Although the variable “Percent HY” had a positive association with the probability of AIV infection in lower-ranked models from which year was excluded, this variable was not retained in the best-supported model. The probability of being infected with AIV was not different among species when all other explanatory variables were considered.

### British Columbia

Candidate models to explain variation in AIV infection status in BC are displayed in S5 Table. Based on the best supported model, infection status was affected by year: ducks were more likely to be infected in 2005 compared to all other years when sampling occurred (Table 3). Males were 1.43 times more likely to be infected than females (CI = 1.04, 1.82) (Table 3). Green-winged teal were less likely to be infected than mallards (OR = 0.38, CI = 0.19, 0.77) (Table 3). The variables age, population density and percent HY could not be examined due to missing data (S2 Table). The effect of temperature and sampling date could not be assessed because of strong associations between these variables, and with sampling location.

**Table 3:** Maximum likelihood estimates of effects in the best-approximating model of AIV infection in BC.

Variable	Term	MLE	SE	95% CI
Intercept		-2.033	0.246	-2.515, -1.551
Sex (ref = Female)	Male	0.354	0.101	0.156, 0.553
Species (ref = MALL)	AGWT	-0.975	0.363	-1.687, -0.264
	AMWI	-0.463	0.498	-1.440, 0.513
	BWTE	-0.679	0.3500	-1.364, 0.007
	NOPI	-1.054	0.437	-1.911, -0.197
Year (ref = 2005)	2006	-1.025	0.126	-1.273, -0.778
	2007	-1.593	0.158	-1.903, -1.283
	2008	-1.228	0.235	-1.688, -0.768
	2010	-0.762	0.257	-1.267, -0.258
Sampling location (ref = Alaksen)	Minnie Lake	2.127	0.235	1.666, 2.587
	Nicola Lake	2.565	0.2300	2.114, 3.015

The model was fitted as a generalized linear model with a sample size of 2281. All values shown are on the logit scale. Exponentiation of estimates provides the odds ratio interpretation of effect size. MLE is the maximum likelihood estimate of the parameter, SE is the standard error of the estimate, and 2.5% and 97.5% values define the 95% confidence interval around the MLE.

Abbreviations: MALL: *A*. *platyrhynchos*, AGWT: *A*. *carolinensis*, AMWI: *A*. *americana*, BWTE: *A*. *discors*, NOPI: *A*. *acuta*

## Discussion

AIV prevalence in wild ducks in Canada was related to several demographic and ecological factors, including age, sex, species, population density, air temperature, and the proportion of HY ducks in the population.

### Population density

Although the role of wild bird population density in partially driving the dynamics of AIV transmission and exposure in waterfowl populations has been frequently hypothesized [51,52,53], there are very few reports which have illustrated a clear link between population density and AIV infection [22,23]. Few studies have quantified waterfowl population density in relation to AIV infection across a large geographic range, largely because of the difficulty in reliably and consistently estimating population sizes at time of sampling, and because of the significant financial and human resources required to do so. We were fortunate to be able to make use of data available through the Waterfowl Breeding Population and Habitat Survey [45], which has been using aerial and ground surveys to estimate population density in key waterfowl breeding areas across Canada every year since 1955. In our study, the positive association between AIV infection and population density is robust because it was observed in both the Prairie Provinces and Eastern Canada (Tables 1 and 2; Fig 2), indicating the importance of this variable despite differences between these two regions in the range of population density values, methods of estimating population density, species composition, sampling dates and geographic characteristics of sampling locations. Likewise, in a study of African ducks, AIV prevalence was positively related to density of wildfowl [22], and AIV prevalence in blue-winged teal was positively associated with relative density of conspecifics in the Prairie Provinces in Canada [23]. By increasing the frequency of contact rates between susceptible birds and infected birds, or between susceptible birds and contaminated fecal material, higher bird densities may increase the probability of transmission and exposure of virus, and may also result in a higher diversity of circulating AIV strains.

Higher population density in the spring could also result in earlier transmission of viruses, accelerating rates of infection during spring, prior to and during hatching of ducklings. Higher densities during development could also accentuate disease transmission among immunologically naïve ducklings of all co-occurring duck species.

### Temperature

Lower temperatures during and preceding the time of sampling were strongly associated with increased probability of AIV infection for dabbling ducks in Eastern Canada (Table 1, Fig 3). Such a temperature effect was not evident in the Prairie Provinces, possibly due to much narrower and warmer temperature ranges observed during the sampling periods in this region (12.3–23.5) compared to Eastern Canada (1.9–23.1). Other studies found associations between low temperature conditions and probability of AIV infection in Asia, Europe and North America [26,27,54]. Low water temperatures have been associated with higher persistence of virus particles in laboratory experiments [24,30]. Mean maximum daily temperature around the time of sampling was not a significant variable in the best-supported models in a study of wild ducks in Africa [22], however mean daily temperature ranges were likely higher in their sites compared to those observed in Canada.

### Sampling time

Our study focused on samples collected in late summer-fall when HY ducks have fledged and large numbers of ducks of all ages aggregate before southward migration. The probability that a duck would test positive for AIV peaked in mid-August in the Prairie Provinces and between August and September in Eastern Canada (Tables 1 and 2, Fig 4).

The observed peak in probability of AIV infection in mid-August in the Prairie Provinces matches the temporal pattern previously reported for dabbling ducks, which has been attributed to both the recruitment of naïve HY ducks and pre-migratory aggregation and associated increase in population density [5,31,32,35]. A more prolonged peak of AIV infection risk was evident in Eastern Canada compared to the Prairie Provinces (Fig 5), perhaps due to a longer pre-migration aggregation period, or due to new migrants from northern breeding locations coming into the population, stopping over on their way south, bringing new viruses into the population, or being newly exposed to local circulating viruses [55]. In Eastern Canada, only American black ducks and mallards were sampled in October-December, suggestive of semi-resident populations of these species in the East which may become immune to local circulating AIV strains by late fall [34]. The weather in Eastern Canada is also typically milder and the breeding period is longer compared to the Prairie Provinces. A prolonged peak of AIV infection risk was also evident in studies in other geographic areas with climatic conditions similar to those in Eastern Canada [33,34,56,57].

### Annual variability

Our models showed annual variability in predicted prevalence of AIV infection, being lowest in 2008 in Eastern Canada and the Prairie Provinces, and in 2007 in BC (Tables 1–3, Fig 5). Given the relatively short time frame of this study period, we were unable to observe a cyclical pattern in annual prevalence. Previous studies in the prairies and elsewhere have suggested a two to four year cycle of AIV prevalence [28,29,35,57], and it is possible that our results for the Prairie Provinces are consistent with this. Longer term studies would be needed to confirm the existence of a cyclical pattern at our sites. Periodicity in prevalence could be the result of infection-immunity cycles in duck populations [35]. However, since we did not have information on the AIV-specific immunological status of ducks in our study, we were unable to test the temporal association between infection and antibody status.

### Age at the individual and population levels

Age affected the probability of infection with AIV at both the individual and population levels (Tables 1 and 2). As reported in other studies, juvenile birds were more likely to be infected than adult birds [23,27,29,32,33,34,35,36,37]. This is probably because young birds are immunologically naïve or have been exposed to fewer strains of AIV. This explanation is supported by the finding that adult birds are more likely to have antibodies against AIV than are HY birds [34,35]. HY birds infected with AIV for the first time shed higher quantities of virus [34,58], and may also clear infection more slowly, and therefore may remain infected with AIV for a longer period of time, allowing for a higher probability of detection [7,58,59,60].

We also observed an effect of age at the population level: the probability of AIV infection was positively associated with the proportion of young birds in the fall in Eastern Canada (Table 1). Although numerous studies have suggested such a correlation indirectly based on the coincidence of a peak in AIV prevalence with the entrance of HY birds into the population [31,34,51], this is the first study to clearly document such an association. Although not being part of the top ranking model, percent HY was also positively associated with probability of AIV infection in the Prairie Provinces, particularly in models that did not contain the variable year, which absorbed much of the annual variation caused by other variables (S4 Table). Thus it is possible that some of the variation explained by year in our top ranking model might have been due to the annual variation in percent HY. These findings are consistent with a hypothesis that higher proportions of immunologically naïve birds in pre-migratory flocks facilitate the spread and shedding of AIV in the population.

### Sex

The scientific literature is inconsistent regarding the probability of AIV infection in relation to sex. Female ducks were more likely to be infected than males in one study [39], while males were more susceptible in others [27,35,36] or there was no difference [61]. In our study, males were more likely to be infected than females in both age groups in Eastern Canada and in HY birds in BC, while AHY females in the Prairie Provinces were more likely to be infected than AHY males. Contradictory results in the literature may arise from interactions between age, sex, and time of sampling. An interaction was evident between age and sex on AIV prevalence in ducks in Alaska [39,40]. An age-sex interaction could relate to differences in life-cycle events, such as time of moult, between AHY males and females and may or may not be present depending on the time of sampling in different regions.

### Species

We found species differences in AIV prevalence in Eastern Canada and BC. Mallards were more likely to be infected than blue- and green-winged teal and American wigeon in Eastern Canada (Table 1) and more than green-winged teal and northern pintail in BC (Table 3). Others have reported that mallards had the highest prevalence of AIV infection among sympatric dabbling ducks [27,32,36,41], while Munster et al. reported higher prevalence in both mallards and teal [57]. In the Prairie Provinces, no such species differences were observed (Table 2). It seems possible that differences in probability of AIV infection among dabbling duck species at any given time and geographical area are partially due to season and the prevalence of infection in the population, in addition to potential intrinsic physiological differences among species with respect to immunity, shedding patterns or adaptation to the virus [62]. Furthermore, transmission of AIVs during the pre-fledge and post-fledge periods in summer was found to occur predominantly within species, with progressively increasing interspecific transmission during fall and winter [63]. Given that most of our samples were collected in the summer, it is possible that species differences in infection were also a reflection of the differences in AIVs circulating within species. Our results do not indicate a special role for mallards in increasing the probability of AIV infection of other local duck populations as speculated by others [27,57], however it is possible that a role for mallards would become more apparent during the fall and winter periods when more interspecific transmission occurs [63].

### Conclusions

In conclusion, we explored a large number of potential variables affecting the prevalence of AIV infection in wild ducks across Canada, based on a large sample of birds, enabling us to make inferences that improve our understanding of the ecology of avian influenza viruses in free-ranging duck populations. One implication from our results pertains to targeting key locations and time periods for AIV surveillance in wild ducks. For instance, waterfowl population densities in the spring may be used to predict locations that will have the highest AIV prevalences months later in late summer and fall. Thus, focusing monitoring efforts in these areas during mid-August (Prairie Provinces) and Aug-Sept (Eastern Canada) when prevalence of infection is highest, while targeting HY birds may be a cost-effective strategy to maximize the probability of detecting and characterizing AIVs circulating in the population. This is particularly relevant due to numerous recent reports of highly pathogenic strains of AIV in North American wild birds beginning in late 2014 to 2016 (H5N8, H5N2) [3,64,65,66,67,68]), ongoing reports of H5N8, H5N6, H5N1, and H7N9 in multiple Eurasian countries [3], and the increased interest in heightened vigilance for morbidity and mortality events in North American wild birds [69].

## Supporting information

S1 Table Predictor variables used in model sets of AIV infection in the Prairie Provinces and Eastern Canada. (DOCX)

S2 Table Number of samples per category of explanatory variables used in statistical analyses. (DOCX)

S3 Table Models fitted to explain variation in AIV infection probability in dabbling ducks sampled in Eastern Canada as part of national surveillance programs from 2005 to 2011. (DOCX)

S4 Table Models fitted to explain variation in AIV infection probability in dabbling ducks sampled in the Prairie Provinces as part of national surveillance programs from 2005 to 2011. (DOCX)

S5 Table Models fitted to explain variation in AIV infection probability in dabbling ducks sampled in British Columbia as part of national surveillance programs from 2005 to 2011. (DOCX)
