# Supplementary material for: The ecology of avian influenza viruses in wild dabbling ducks (Anas spp.) in Canada
Source: PLoS One. 2017 May 5;12(5):e0176297. doi: 10.1371/journal.pone.0176297 (PMC5419510; doi:10.1371/journal.pone.0176297)
Supplement: S1 Table — (DOCX) [file pone.0176297.s001.docx]

S1 Table Predictor variables used in model sets of AIV infection in the Prairie Provinces and Eastern Canada

| **Fixed effect variables** | **Type** | **Definition** | **Range or Description** |
| --- | --- | --- | --- |
| Age | Categorical | Age of the birds at the time of capture | Hatch year (HY), after hatch year (AHY) |
| Sex | Categorical | Gender of the birds | female, male |
| Species | Categorical | *Anas* spp. | Mallard (*A. platyrhynchos*), American black duck (*A. rubripes*) and mallard-black duck hybrids, blue-winged teal (*A. discors*), green-winged teal (*A. carolinensis*), northern pintail (*A. acuta*), American wigeon (*A. americana*) and gadwall (*A. strepera*).* |
| Population density | Numerical | Spring breeding population density (all dabbling ducks) | Total number of ducks/km^2^ ranged between 2.15- 11.58 in the Prairie Provinces, and 0.33- 2.87 in Eastern Canada. |
| Pond density | Numerical | Spring pond density | 6.0-7.6 ponds/ km^2^ in the Prairie Provinces. |
| Percent HY | Numerical | Percentage of HY ducks in the harvest population | 67-93% (Eastern Canada), 49-83% in Prairie Provinces |
| Percent MALL | Numerical | Percent of mallards in the breeding population of all dabbling ducks | 22.7-56.55 % in the Prairie Provinces and 0.12-90 % in Eastern Canada |
| Temperature | Numerical | Average of mean daily temperature over two weeks before the sampling day (sampling day inclusive) | 12.3-23.5 ^o^C in the Prairie Provinces and 1.9-23.1in Eastern Canada |
| Sampling time | Categorical | Approximately 2-week periods of sampling** | Between July 23 – Sept 1 in the Prairie Provinces, July 21 – Dec 30 in Eastern Canada and Aug 2 - Dec 8 in BC |
| Year | Categorical | Calendar year of sampling | 2005-2011 |
|  |  |  |  |
| **Random variable ***** |  |  |  |
| Sampling location | Categorical | Lake/wetland where sampling occurred | 20 sites in the prairies, 72 in eastern Canada |

* Gadwall samples from Eastern Canada and BC, and black duck samples from the Prairie Provinces and BC were excluded because of too few samples and no AIV positives.

** Because of fewer samples in the fall and winter months, the month of September, the month of October and the combined months of Nov-Dec constitute one period each in Eastern Canada. The first three time periods approximately match those in the Prairie Provinces.

*** Sampling locations in British Columbia were not included as part of the random variable, but as fixed effects.
