# Supplementary material for: The ecology of avian influenza viruses in wild dabbling ducks (Anas spp.) in Canada
Source: PLoS One. 2017 May 5;12(5):e0176297. doi: 10.1371/journal.pone.0176297 (PMC5419510; doi:10.1371/journal.pone.0176297)
Supplement: S2 Table — (DOCX) [file pone.0176297.s002.docx]

**S2 Table.** Number of samples per category of explanatory variables used in statistical analyses.

| Variable | Categories | East | Prairies | BC | Total |
| --- | --- | --- | --- | --- | --- |
| Age | HY | 7484 | 4255 | 2278 | 14017 |
|  | AHY | 1483 | 3654 | 3 | 5140 |
| Sex | female | 3767 | 2694 | 1077 | 7538 |
|  | male | 5200 | 5215 | 1204 | 11619 |
| Species | MALL *(A. platyrhynchos)* | 3596 | 3804 | 2049 | 9449 |
|  | ABDU *(A. rubripes)* | 2829 | 0 | 0 | 2829 |
|  | BWTE *(A. discors)* | 1226 | 2996 | 40 | 4262 |
|  | AGWT *(A. carolinensis)* | 965 | 67 | 46 | 1078 |
|  | NOPI *(A. acuta)* | 67 | 938 | 29 | 1034 |
|  | AMWI *(A. americana)* | 284 | 31 | 117 | 432 |
|  | GADW *(A. strepera)* | 0 | 73 | 0 | 73 |
| Year | 2005 | 2030 | 1084 | 596 | 3710 |
|  | 2006 | 1555 | 803 | 1177 | 3535 |
|  | 2007 | 2375 | 2394 | 327 | 5096 |
|  | 2008 | 374 | 990 | 103 | 1467 |
|  | 2009 | 1026 | 880 | 0 | 1906 |
|  | 2010 | 1343 | 947 | 78 | 2368 |
|  | 2011 | 264 | 811 | 0 | 1075 |
| Total | All categories | 8967 | 7909 | 2281 | 19157 |

Abbreviations:

HY = hatch year, AHY = after hatch year

MALL: Mallard (*A. platyrhynchos*)

ABDU: American black duck (*A. rubripes*) and mallard-black duck hybrids

BWTE: blue-winged teal (*A. discors*)

GWTE: green-winged teal (*A. carolinensis*)

NOPI: northern pintail (*A. acuta*)

AMWI: American wigeon (*A. americana*)

GADW: gadwall (*A. strepera*)
