# Supplementary material for: The ecology of avian influenza viruses in wild dabbling ducks (Anas spp.) in Canada
Source: PLoS One. 2017 May 5;12(5):e0176297. doi: 10.1371/journal.pone.0176297 (PMC5419510; doi:10.1371/journal.pone.0176297)
Supplement: S3 Table — (DOCX) [file pone.0176297.s003.docx]

**S3 Table.** Models fitted to explain variation in AIV infection probability in dabbling ducks sampled in Eastern Canada as part of national surveillance programs from 2005 to 2011 (n = 8967). Sampling location was included as random effect. *Variables in italics were non informative (inclusion in the model increased AICc).*

| Model | k | AICc | ΔAICc | loglik |
| --- | --- | --- | --- | --- |
| **Age, Sex, Species, Year, Sampling Time, Population density, Percent HY, Temperature** | 22 | 8854.9 | 0.0 | -4404.4 |
| Age, Sex, *Age*Sex,* Species, Year, Sampling Time, Population Density, Percent HY, Temperature | 23 | 8856.9 | 2.0 | -4404.4 |
| Age, Sex, *Age*Sex,* Species, Year, Sampling Time, Percent HY, Temperature | 22 | 8862.0 | 7.1 | -4407.9 |
| Age, Sex, *Age*Sex,* Species, Year, Sampling Time, Population density, Temperature | 22 | 8865.6 | 10.7 | -4409.7 |
| Age, Sex, *Age*Sex,* Species, Year, Sampling Time, Temperature | 21 | 8867.8 | 12.9 | -4411.8 |
| Age, Sex, *Age*Sex,* Species, Year, Sampling Time, Population density, Percent HY | 22 | 8870.0 | 15.1 | -4411.9 |
| Age, Sex, *Age*Sex,* Species, Year, Sampling Time, Percent HY | 21 | 8873.4 | 18.5 | -4414.6 |
| Age, Sex, *Age*Sex,* Species, Year, Sampling Time, Population density | 21 | 8878.4 | 23.6 | -4417.2 |
| Age, Sex, *Age*Sex,* Species, Year, Sampling Time | 20 | 8879.3 | 24.4 | -4418.6 |
| Age, Sex, *Age*Sex,* Species, Year | 15 | 8923.3 | 68.4 | -4445.6 |
| Year | 7 | 9024.2 | 169.3 | -4504.1 |
| Age, Sex, Species | 8 | 9404.3 | 549.5 | -4693.2 |
| Age, Sex, *Age*Sex,* Species | 9 | 9406.1 | 551.2 | -4693.0 |
| Age, Sex | 3 | 9495.7 | 640.8 | -4743.8 |
| Age, Sex, *Age*Sex* | 4 | 9497.3 | 642.4 | -4743.7 |
| Age | 2 | 9500.4 | 645.5 | -4747.2 |
| Species | 6 | 9531.2 | 676.3 | -4758.6 |
| Percent HY | 2 | 9555.1 | 700.2 | -4774.5 |
| Sampling Time | 6 | 9619.2 | 764.4 | -4802.6 |
| Population Density | 2 | 9630.6 | 775.7 | -4812.3 |
| Temperature | 2 | 9638.0 | 783.1 | -4816.0 |
| Sex | 2 | 9638.6 | 783.7 | -4816.3 |
| Null (random intercept only: sampling site) | 1 | 9642.8 | 787.9 | -4819.4 |
| *Percent MALL* | 2 | 9644.0 | 789.2 | -4819.0 |

k= number of parameters in the model

AIC_c_ = Akaike's Information Criterion adjusted for small sample size

ΔAIC_c_ = difference between AIC_c_  values of the best supported model and the given model

loglik: the natural logarithm of the likelihood function
