# Supplementary material for: The ecology of avian influenza viruses in wild dabbling ducks (Anas spp.) in Canada
Source: PLoS One. 2017 May 5;12(5):e0176297. doi: 10.1371/journal.pone.0176297 (PMC5419510; doi:10.1371/journal.pone.0176297)
Supplement: S4 Table — (DOCX) [file pone.0176297.s004.docx]

**S4 Table.** Models fitted to explain variation in AIV infection probability in dabbling ducks sampled in the Prairie Provinces as part of national surveillance programs from 2005 to 2011 (n = 7909). Sampling location was included as random effect. *Variables in italics are non informative (inclusion in the model increases AIC).*

| Model | k | AICc | ΔAICc | loglik |
| --- | --- | --- | --- | --- |
| **Age, Sex, Age*Sex, Year, Sampling Time, Population density** | 13 | 6325.1 | 0.0 | -3148.5 |
| Age, Sex, Age*Sex, *Species*, Year, Sampling Time, Population density | 18 | 6328.4 | 3.3 | -3145.2 |
| Age, Sex, Age*Sex, *Species,* Year, Sampling Time, Population density, *Percent HY* | 19 | 6330.4 | 5.3 | -3145.2 |
| Age, Sex, Age*Sex, *Species,* Year, Sampling Time, Population density, *Percent HY, Temperature* | 20 | 6332.2 | 7.1 | -3145.1 |
| Age, Sex, Age*Sex, *Species,* Year, Sampling Time, Population density, *Percent HY, Temperature, Percent MALL* | 21 | 6334.1 | 9.0 | -3145.0 |
| Age, Sex, Age*Sex, *Species*, Year, Sampling Time, Percent MALL | 18 | 6334.6 | 9.5 | -3148.2 |
| Age, Sex, Age*Sex, *Species*, Year, Sampling Time, Percent HY | 18 | 6337.4 | 12.3 | -3149.7 |
| Age, Sex, Age*Sex, *Species*, Year, Sampling Time | 17 | 6338.1 | 13.0 | -3151.0 |
| Age, Sex, Age*Sex, *Species,* Year, Sampling Time, *Temperature* | 18 | 6338.7 | 13.6 | -3150.3 |
| Age, Sex, Age*Sex, *Species,* Year | 15 | 6399.7 | 74.6 | -3183.8 |
| Age, Sex, Age*Sex, Species | 9 | 6769.3 | 444.2 | -3374.6 |
| Age, Sex, Age*Sex | 4 | 6771.7 | 446.5 | -3380.8 |
| Age | 2 | 6776.1 | 451.0 | -3385.1 |
| Age, *Sex* | 3 | 6777.8 | 452.7 | -3384.9 |
| Percent HY | 2 | 6982.0 | 656.9 | -3488.0 |
| Species | 6 | 7118.8 | 793.7 | -3552.4 |
| Temperature | 2 | 7140.3 | 815.2 | -3567.2 |
| Sampling Time | 3 | 7148.1 | 823.0 | -3570.1 |
| Sex | 2 | 7152.7 | 827.6 | -3573.4 |
| Percent MALL | 2 | 7159.5 | 834.4 | -3576.7 |
| Population Density | 2 | 7165.2 | 840.1 | -3579.6 |
| Null (random intercept: sampling_site) | 1 | 7166.5 | 841.4 | -3581.3 |
| *Pond density* | 2 | 7168.3 | 843.2 | -3581.2 |

K = number of parameters in the model

AIC_c_ = Akaike's Information Criterion adjusted for small sample size

ΔAIC_c_ = difference between AIC_c_ values of the best supported model and the given model

loglik: the natural logarithm of the likelihood function
