# Supplementary material for: The ecology of avian influenza viruses in wild dabbling ducks (Anas spp.) in Canada
Source: PLoS One. 2017 May 5;12(5):e0176297. doi: 10.1371/journal.pone.0176297 (PMC5419510; doi:10.1371/journal.pone.0176297)
Supplement: S5 Table — (DOCX) [file pone.0176297.s005.docx]

**S5 Table.** Models fitted to explain variation in AIV infection probability in dabbling ducks sampled in British Columbia as part of national surveillance programs from 2005 to 2011 (n = 2281). Sampling location was included as a fixed effect in combination with other variables.

| Model | k | AICc | ΔAICc | loglik |
| --- | --- | --- | --- | --- |
| Sampling Location, Sex, Species, Year | 12 | 2434.2 | 0.0 | -1205.0 |
| Sampling Location, Sex, Year | 8 | 2443.9 | 9.7 | -1213.9 |
| Sampling Location, Species, Year | 11 | 2444.6 | 10.4 | -1211.2 |
| Sampling Location, Year | 7 | 2454.2 | 20.0 | -1220.1 |
| Sampling Location, Sex, Species | 8 | 2557.8 | 123.6 | -1270.8 |
| Sampling Location, Sex | 4 | 2559.8 | 125.6 | -1275.9 |
| Sampling Location, Species | 7 | 2561.9 | 127.7 | -1273.9 |
| Sampling Location | 3 | 2563.8 | 129.6 | -1278.9 |
| Year | 5 | 2683.8 | 249.6 | -1336.9 |
| Species | 5 | 2849.5 | 415.3 | -1419.8 |
| Sex | 2 | 2885.3 | 451.2 | -1440.7 |
| null | 1 | 2908.1 | 473.9 | -1453.0 |

k= number of parameters in the model

AIC_c_ = Akaike's Information Criterion adjusted for small sample size

ΔAIC_c_ = difference between AIC_c_ values of the best supported model and the given model

loglik: the natural logarithm of the likelihood function
